# Proinflammatory Cytokines Are Soluble Mediators Linked with Ventricular Arrhythmias and Contractile Dysfunction in a Rat Model of Metabolic Syndrome

**DOI:** 10.1155/2017/7682569

**Published:** 2017-10-19

**Authors:** Evaristo Fernández-Sada, Alejandro Torres-Quintanilla, Christian Silva-Platas, Noemí García, B. Cicero Willis, César Rodríguez-Rodríguez, Erasmo De la Peña, Judith Bernal-Ramírez, Niria Treviño-Saldaña, Yuriana Oropeza-Almazán, Elena C. Castillo, Leticia Elizondo-Montemayor, Karla Carvajal, Gerardo García-Rivas

**Affiliations:** ^1^Cátedra de Cardiología, Escuela de Medicina, Tecnológico de Monterrey, Monterrey, Mexico; ^2^Centro de Investigación Biomédica, Hospital Zambrano Hellion, Tec Salud del Sistema Tecnológico de Monterrey, Monterrey, Mexico; ^3^Centro de Investigación en Nutrición Clínica y Obesidad, Escuela de Medicina, Tecnológico de Monterrey, Monterrey, Mexico; ^4^Laboratorio de Nutrición Experimental, Instituto Nacional de Pediatría, Ciudad de México, Mexico

## Abstract

Metabolic syndrome (MS) increases cardiovascular risk and is associated with cardiac dysfunction and arrhythmias, although the precise mechanisms are still under study. Chronic inflammation in MS has emerged as a possible cause of adverse cardiac events. Male Wistar rats fed with 30% sucrose in drinking water and standard chow for 25–27 weeks were compared to a control group. The MS group showed increased weight, visceral fat, blood pressure, and serum triglycerides. The most important increases in serum cytokines included IL-1*β* (7-fold), TNF-*α* (84%), IL-6 (41%), and leptin (2-fold), the latter also showing increased gene expression in heart tissue (35-fold). Heart function ex vivo in MS group showed a decreased mechanical performance response to isoproterenol challenge (ISO). Importantly, MS hearts under ISO showed nearly twofold the incidence of ventricular fibrillation. Healthy rat cardiomyocytes exposed to MS group serum displayed impaired contractile function and Ca^2+^ handling during ISO treatment, showing slightly decreased cell shortening and Ca^2+^ transient amplitude (23%), slower cytosolic calcium removal (17%), and more frequent spontaneous Ca^2+^ release events (7.5-fold). As spontaneous Ca^2+^ releases provide a substrate for ventricular arrhythmias, our study highlights the possible role of serum proinflammatory mediators in the development of arrhythmic events during MS.

## 1. Introduction

Obesity is unquestionably an important health problem worldwide. The WHO states that obesity is no longer a problem for high-income countries alone, and it has been present in low- and middle-income nations, including urban locations in Latin America and North Africa, since the 1980s [1]. Current estimates indicate that more than one-third of the world's population is overweight or obese [2]. In Mexico and the United States, more than one-third of the adult population is overweight, and more than one-third is obese [3, 4]. Widespread obesity and metabolic syndrome (MS) are strongly related to a shift in diet towards energy-dense foods rich in sugars and fats [5], and these conditions have been linked to adverse cardiovascular prognosis and mortality [6]. In addition, obesity and MS are associated with an increased risk of sudden cardiac death. Worldwide studies show that patients with obesity have approximately twice the risk of sudden death and that patients with diabetes have three times the risk of age-matched controls [7]. Indeed, circulating fatty acid levels have been identified as a risk factor for sudden death independently of myocardial infarction [8]. Despite the increasing prevalence of obesity and MS, we have a limited understanding of the contribution of metabolic abnormalities to arrhythmogenic events. One interesting observation is that higher levels of proinflammatory cytokines in patients with obesity correlate with sudden cardiac death [9]. Of note, during the obesogenic state, adipose tissue promotes an increase in serum inflammatory cytokines, such as interleukin-6 (IL-6) and tumor necrosis factor-*α* (TNF-*α*), in different tissues like fat and skeletal muscle, leading to metabolic abnormalities [10, 11]. Furthermore, prior work with obese and/or MS animal models has implicated proinflammatory cytokine production with macrophage infiltration in skeletal muscle leading to insulin resistance [12], and their production has also been detected in the pancreas and the heart [13].

A previous study reported that obesity induced by a high fat diet (HFD) in mice leads to increased cardiac and serum IL-6 levels, along with myocardial disruption of glucose metabolism [14]. Furthermore, high levels of proinflammatory cytokines, including TNF-*α*, IL-6, IL-1*β*, and IL-2, play a major role in the pathogenesis and prognosis of ventricular dysfunction [15–19]. In this regard, several proinflammatory cytokines modulate membrane potential and Ca^2+^ handling. For instance, IL-1*β* and TNF-*α* induce abnormal Ca^2+^ homeostasis and arrhythmogenicity in ventricular cardiomyocytes [20]. TNF-*α* can also decrease the expression of SERCA2, which consequently prolongs the Ca^2+^ transient duration and action potentials [20]. Furthermore, ventricular cardiomyocytes treated with TNF-*α* and IL-1*β* have increased vulnerability to sarcoplasmic reticulum Ca^2+^ leak and are prone to spontaneous Ca^2+^ release (SCR) events [20]. Moreover, isolated ventricular cells from prediabetic rats showed that reactive oxygen species (ROS) are also involved in the higher SCR and rise of diastolic Ca^2+^, promoting spontaneous ventricular contractions and arrhythmogenesis *in vivo*, possibly due to increased ryanodine receptor (RyR) phosphorylation by the hyperactivation of oxidized Ca^2+^—calmodulin Kinase II (CaMKII) [21]. Finally, obesity is known to increase the risk of atrial fibrillation in men and women [22]. However, it is not clear how much of the risk of arrhythmogenesis and sudden cardiac death is attributable to a chronic proinflammatory status in patients with obesity and MS. Here, we sought to explore whether arrhythmogenic events associated with MS can be triggered by serum mediators of chronic inflammation and not just metabolic disturbance. Accordingly, we analyzed whether contractile dysfunction, impaired Ca^2+^ handling, and spontaneous Ca^2+^ releases were reproduced by exposing healthy cardiac cells to MS serum.

## 2. Materials and Methods

### 2.1. Animals

All the experiments were performed in accordance to the animal care guidelines from the Guide for the Care and Use of Laboratory Animals published by the US National Institutes of Health (NIH Publication number 85–23, revised 1996). All procedures were approved by the animal use and care committee from the medical school at Tecnológico de Monterrey (Project number 2011–02). Sex- and age-matched male Wistar rats weighing 200–230 g were used for all the experiments. Two experimental groups were assigned: (1) control group drinking tap water and (2) MS group receiving 30% refined commercial sugar in drinking water during 25–27 weeks, as previously described [23, 24]. Both groups were fed with standard rat chow diet *ad libitum*. At the end of the treatment period, blood samples were obtained from both groups by tail venous access and serum was separated by centrifugation. Triglyceride levels were determined using Liquicolor GPO-PAP (Stanbio Laboratory). Serum cytokine levels were determined by commercial ELISA kits (PeproTech), following experimental protocols provided by the manufacturer. TNF-*α*, IL-6, IL-1*β*, IL-10, and leptin serum levels were determined in triplicate for each sample. Cytokines and cytokine receptor gene expression were analyzed as follows: Total RNA from adipose and cardiac tissue was isolated using the TRIzol Reagent (Invitrogen) for cDNA synthesis and subsequent real-time PCR analysis (qPCR) of cytokines, and their receptors were performed using the Rotor-Gene 3000 (Corbett Research). All samples were subjected to 0.5 *μ*L of DNAse treatment (Agilent Technologies Inc., Santa Clara, California, USA), and cDNA was synthesized from 1.0 *μ*g total RNA using the AffinityScript cDNA Synthesis Kit (Agilent Technologies). Actin-*β* (actb) was used as an endogenous control to allow the relative quantification of the genes of interest. The qPCR was performed with the Brilliant III Ultra-Fast SYBR Green QPCR Master Mix (Agilent Technologies) on both targets and the endogenous control (the probes used are shown in Supplemental Table 1 in Supplementary Material available online at https://doi.org/10.1155/2017/7682569). The amplified PCR products were quantified by measuring the calculated cycle thresholds (C_T_). The relative expression of specific mRNA in the samples was calculated by the ΔΔC_T_ method. The mean value of the control group target levels was used as the calibrator (one per sample), and the results were expressed as the difference of the relative expression level data (2^−ΔΔCT^) relative to the control group.

### 2.2. Computerized Axial Tomography

Body composition of adipose tissue and skeletal muscle were analyzed in the control and the MS rats by using whole-body computer axial tomography (CAT) scans. In brief, the animals were sedated with pentobarbital (40 mg/kg i.p.) and images were obtained by using a Siemens tomograph with 16 detectors. The variables for image acquisition were as follows: Kv: 180, mA: 262, rot: 0.5 s, thickness: 1.0 mm, sp filter: 1.0, and tilt: 0.0°. A cardiovascular imaging specialist who was blinded from the group classification did the image analysis. Data was obtained with 4 rats for each group. The region of interest was manually selected. A Hounsfield unit threshold (−250 to 120) was applied to eliminate air and bone. The remaining tissues were calculated by means of a 3-colored map with the following Hounsfield unit ranges: −250–0: adipose tissue, 0–20: water, and 20–90: skeletal muscle and organs.

### 2.3. Echocardiography

To evaluate cardiac function, transthoracic echocardiography was performed as previously described [25]. To avoid subjective interpretation, all studies were performed and analyzed by the same trained person. In brief, the animals were placed in supine and left lateral decubitus position and a Philips EnVisor® echocardiogram was used to measure transmitral flow (E wave and A wave) and fractional shortening (FS = diastolic LVID—systolic LVID/diastolic LVID × 100 [LVID, LV internal diameter]). Transmitral flow velocities were obtained by positioning a pulsed Doppler wave sample volume at the tip of the mitral valve leaflets during diastole in the apical-four chamber view. The early (E) and late (A) diastolic waves were measured. The internal diameter of the LV was calculated by tracing the endocardial border during maximum contraction and relaxation in the parasternal long axis view.

### 2.4. Isolated Heart Studies

To analyze cardiac function, hearts were isolated as described previously [26] with minor modifications. Male Wistar rats (250–300 g) were anesthetized with pentobarbital (80 mg/kg, i.p.) with previous administration of 1000 U/kg of heparin; once corneal reflex was absent bilaterally, the hearts were excised, trimmed of excess tissue, weighed, and rapidly immersed in cold (4°C) Ca^2+^-free Krebs–Henseleit buffer. The hearts were mounted in accordance with the Langendorff model and perfused with Krebs–Henseleit (K-H) buffer (in mM): NaCl 125, KCl 5.4, MgCl2 1.0, NaH_2_PO4 0.5, NaHCO_3_ 25, CaCl2 2.5, glucose 11, and octanoate 0.1, as described previously [27]. Once autonomous contractions were established, a latex balloon filled with saline solution and connected to a pressure transducer was inserted into the left ventricle. Basal arterial pressure was set at 60 mmHg. Data analysis for continuous recording of heart rate, left ventricular pressure (LVP), and maximum positive and negative derivative of LV pressure (+dP/dt and −dP/dt) was performed using data Trax software (WPI). Baseline was established during 5 minutes of K-H perfusion and was followed by perfusion of isoprenaline (ISO) (10 nM). MVO2 and the mechanical performance index (MPI) were obtained from the last 3 min of contraction at each ISO concentration. MPI was defined as the product of LV-developed pressure × HR (LVDP × HR; mmHg × heart beats × min^−1^). For ECG recording, electrodes were placed in the chamber in close approximation with the right atrium for the negative electrode and the apex of the left ventricle for the positive electrode. ECG data were collected using data Trax software at a sampling rate of 2.0 kHz. The ECG from the last 30 sec of each 2 min period was averaged using the advanced ECG analysis module of the program, and the RR interval, heart rate, PR interval, and the QT interval were measured. The traces were manually assessed for arrhythmic events according to the Lambeth Conventions [28] as previously reported [29]. At the end of the stimulation, the hearts were released by cutting through the aorta and were immediately frozen with liquid nitrogen. Finally, the hearts were weighted and stored at −80°C. Phosphocreatine levels were carried out with a dual-pump gradient HPLC as previously described [30]. Standard solutions were prepared in 0.1 M KH_2_PO_4_, pH 7.0, and stored at −80°C to minimize the degradation of phosphocreatine. The standard curves were subjected to linear regression analysis, and calibration factors were determined.

### 2.5. Isolation of Cardiomyocytes and Ca^2+^ Handling Experiments

Ca^2+^ transients and cell shortening were measured as previously described [30]. Isolated adult cardiomyocytes, on laminin-covered glass coverslips, were maintained in M199 medium supplemented with taurine (5 mM), sodium pyruvate (2.5 mM), creatine (5 mM), carnitine (2 mM), HEPES (15 mM), sodium bicarbonate (26.2 mM), and 1% (*v*/*v*) penicillin-streptomycin at 37°C. For all experiments, cells were plated at a density of 30,000 cells/cm^2^ and incubated for 24 hours in supplemented M199 medium with 20% serum from MS or control rats. Later, cardiomyocytes were incubated with Fluo-4 AM (5 *μ*M, Life Technologies) for 45 min to evaluate Ca^2+^ transients. Afterwards, the cells were washed with fluorophore-free solution. Dye-loaded cells were mounted on a perfusion chamber. All fluorescence measurements were acquired with a Leica TCS SP5 confocal microscope equipped with a D-apochromatic 63x, 1.2 NA, and oil objective (Leica Microsystems). An argon laser was used to excite the fluorophore at 488 nm and emission collected at 500–600 nm. Linescan images were recorded along the longitudinal axis of the cell at 400 Hz, with a pixel size of 100 nm. For Ca^2+^ transients and cell shortening, a pinhole optimized for a resolution of 0.4 *μ*m in the focal plane and 1 *μ*m in the *z*-axis was used. For Ca^2+^ transients, cells were field stimulated at 1 Hz (MYP100 MyoPacer). Fluorescence data was normalized as *F*/*F*_0_, where *F* is fluorescence intensity and *F*_0_ is average fluorescence at rest.

### 2.6. Statistical Analysis

The data presented here was analyzed using Student's *t*-test for unpaired data. Data is expressed as mean ± standard error of the mean (SEM). Data processing, statistical tests, and graphs were carried out with Prism 2.0 and Stat Calc from GraphPad Software and Microcal Origin 6.0. ^∗^*p* ≤ 0.05 was considered significant.

## 3. Results

### 3.1. MS Rats Show Intra-Abdominal Accumulation of Adipose Tissue

**Figure 1:**
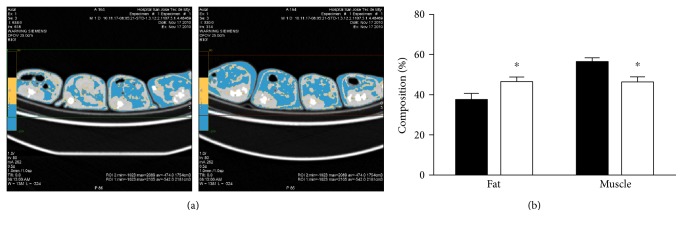
CAT scan analysis of body composition shows higher fat content and reduced muscle mass. (a) Representative image of axial plane from 4 specimens per group. Adipose tissue −250 to 0 Hounsfield units (blue). Water, 0 to 20 (yellow). Skeletal muscle, 20 to 90 (gray). (b) Pooled data for each group showing tissue distribution % from fat and muscle (black, control group; white, MS group). ^∗^*p* < 0.05 versus control. Control group: *n* = 4; MS group: *n* = 4.

We used the MS rat model to explore the role of visceral fat accumulation leading to systemic inflammation and its effect on cardiac function. Body composition parameters as well as mean arterial pressure and serum triglycerides from each group are shown in Table 1. Sucrose-rich drinking water increased total body weight without affecting heart size. However, sugar consumption increased the content of intra-abdominal fat by 2.62-fold when compared with normal diet. Analysis of whole body content of adipose tissue and skeletal muscle by computerized tomography showed a 24% increase in global adipose tissue and an 18% reduction in skeletal muscle mass in MS when compared to control rats (Figure 1). These results show that sugar diet promotes accumulation of intra-abdominal adipose tissue and a relative reduction of muscle mass without affecting heart size.

### 3.2. Sucrose Diet Induces Inflammatory State in Rats

**Figure 2:**
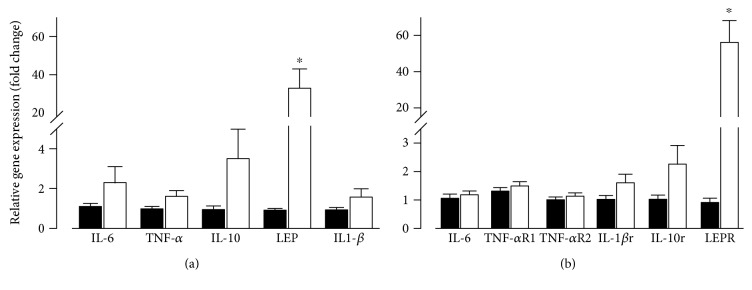
Gene expression of cytokines and their receptors from heart tissue show important increase in leptin and its receptor: pooled data for gene expression from heart tissue for cytokines (a) and corresponding receptors (b). (Black, control group; white, MS group) ^∗^*p* < 0.05 versus control. Control group: *n* = 6–8; MS group: *n* = 6–8.

Serum biochemistry analysis showed a 71% increase in plasma triglycerides and in circulating levels of TNF-*α* (84%), IL-1*β*, IL-6, and leptin (7.7-, 1.4-, and 2-fold) in the MS group when compared to the control group (Table 2). Anti-inflammatory cytokine IL-10 levels did not change significantly. These results showed that sucrose diet promotes accumulation of intra-abdominal fat, associated with higher circulating levels of proinflammatory cytokines. To explore the contribution of cytokine production by the heart, we explored the expression of cytokines and their receptors in cardiac tissue. Gene expression analysis of inflammatory cytokines showed a 35-fold increase in leptin gene expression in cardiac tissue (Figure 2(a)). No changes were found for additional inflammatory markers including TNF-*α*, IL-1*β*, IL-6, and IL-10. When we looked at the gene expression of cytokine receptors, including IL-6, TNFR1, TNFR2, IL-1*β*, and IL-10, no changes were detected in the heart (Figure 2), except for the leptin receptor (35-fold increment). These results show a substantial activation of leptin gene expression in the heart under a sucrose diet. Importantly, IL-6 gene expression in visceral adipose tissue increased 8-fold, which could contribute to the high plasma levels found (Supplemental Figure 1).

### 3.3. MS Rats Show Impaired Cardiac Function during Adrenergic Stimulation

**Figure 3:**
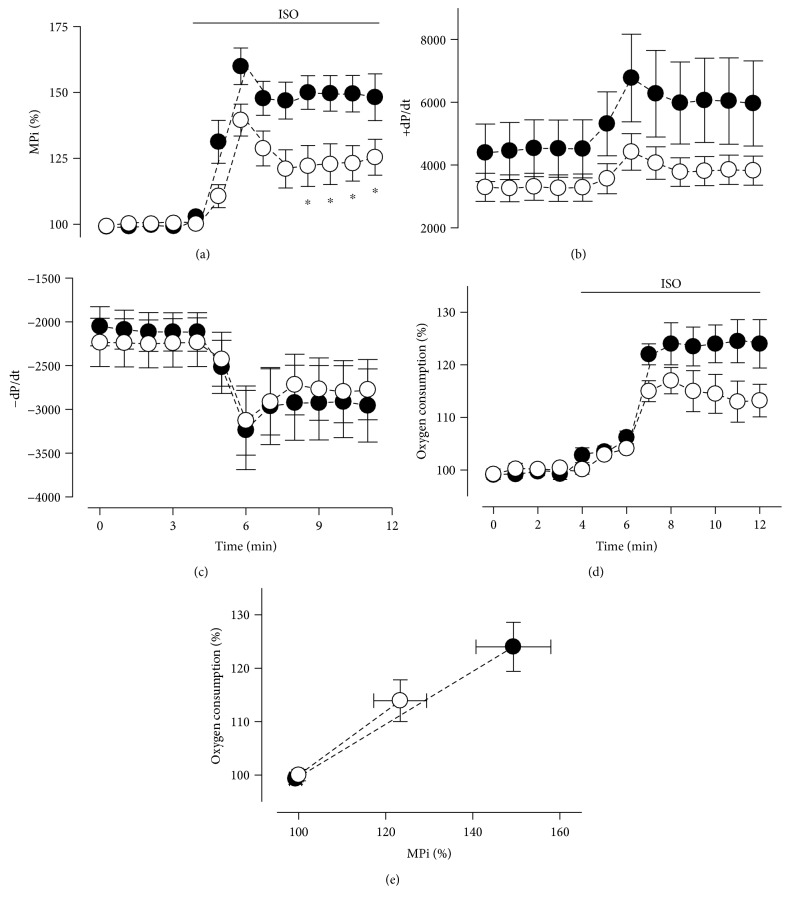
Decreased response to physiological *β*-adrenergic stimulation in hearts from MS rats: Temporal responses to ISO stimulation (10 nM) for (a) normalized mechanical performance index (MPI), (b) maximum positive and (c) negative derivatives of left ventricular pressure (LVP), and (d) normalized oxygen consumption rate (MVO2). (e) Relationship between normalized MPI versus normalized MVO2. (Black, control group; white, MS group) ^∗^*p* < 0.05 versus control. Control group: *n* = 8; MS group: *n* = 9.

Cardiac function was initially evaluated by echocardiography in control and MS rats. A slight impairment in diastolic function in MS rats is shown in Supplemental Table 2. Our ex vivo results showed that basal mechanical performance index (MPi) and oxygen consumption rate (MVO2) of the heart were unaltered in both MS rats and controls (Figure 3(a)). However, *β*-adrenergic stimulation using 10 nM ISO revealed a significantly lower (20%) MPi response in the MS group than in the control group (Figure 3(a)). Accordingly, maximal rate of O2 uptake during ISO stimulation was lower in the MS group compared to control rats, although this was nonsignificant (Figure 3(d)). Of importance, MPi and MVO2 linear correlation was barely altered (Figure 3(e)), suggesting impairment at the adrenergic response level.

Mechanical properties of the MS rats and control hearts were calculated by addressing systolic and diastolic function (+dP/dt and −dP/dt). For systolic function, MS hearts showed an important reduction of contraction rate (4390 ± 916 versus 3289 ± 446 mmHg × s^−1^), demonstrating further decline under *β*-adrenergic stimulation (6038 ± 1376 versus 3848 ± 467 mmHg × s^−1^) (Figure 3(b)). On the other hand, MS hearts showed no important changes in diastolic function at basal conditions or under *β*-adrenergic stimulation using ISO (Figure 3(c)). These findings suggest that MS hearts develop a suboptimal response to *β*-adrenergic stimulation. In addition, we observed that MS hearts also showed a reduction of the phosphocreatine levels during ISO treatment (59 ± 7.5 versus 43 ± 4.3 nmol × mg^−1^; *p* = 0.01). This result reinforces the idea of impaired cardiac function. The observed alterations in MVO2 and phosphocreatine content could be due to deficient mitochondrial oxidative metabolism. In accordance, a similar model of MS identified mitochondrial swelling and mitochondrial membrane depolarization [31].

### 3.4. MS Hearts Are Prone to Arrhythmias

**Figure 4:**
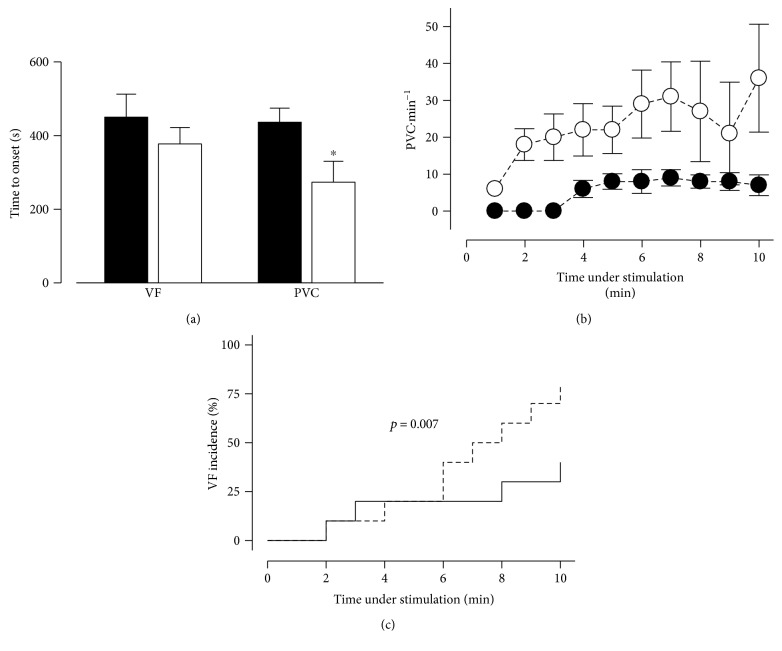
MS increases susceptibility to ventricular arrhythmias. (a) Pooled data for time to onset from ventricular fibrillation (VF) and premature ventricular contractions (PVC). (b) Number of PVCs per minute during ISO stimulation. (c) Incidence of VF. (Black, control group; white and dashed line, MS group) ^∗^*p* < 0.05 versus control. Control *n* = 8; MS *n* = 9.

Afterwards, we found that MS hearts were more prone to developing deadly arrhythmias when maintained under *β*-adrenergic stimulation. The time to onset of premature ventricular contractions (PVC) was 35% shorter (436 s versus 273 s) (Figure 4(a)), and the frequency of PVC was three times higher during most of the time of the adrenergic stimulation for the MS hearts (Figure 4(b)). Ventricular fibrillation had a nearly twofold incidence in MS hearts after 10 minutes of adrenergic stimulation when compared with control hearts (40% versus 73%) (Figure 4(c)). These results suggest that sugar-induced MS promotes cardiac dysfunction, which might be fatal under sustained adrenergic stimulation due to the increased incidence of ventricular arrhythmias. In accordance with this, Sommese et al. observed, in a murine model of MS, diverse ECG alterations such as atrial fibrillation, PVC, sustained ventricular tachycardia, and ventricular fibrillation after caffeine plus epinephrine challenge [21].

### 3.5. Exposure to Serum from MS Rats Increases Cytosolic Ca^2+^ Overload and Spontaneous Ca^2+^ Release (SCR) Events in Cardiac Myocytes

**Figure 5:**
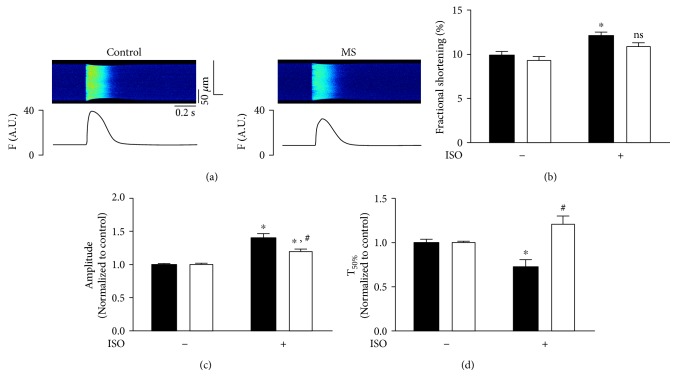
Cytosolic Ca^2+^ transients of control cardiomyocytes after 24 hrs of exposure to MS rat serum. Ca^2+^ transients were recorded for cells from healthy rats after 24 hrs of exposure to control rat serum (control) or MS rat serum (MS), under basal conditions and upon *β*-adrenergic stimulation (100 nM ISO). (a) Representative recording of Ca^+2^ transient for both groups. (b) Pooled data for fractional shortening (%). (c) Pooled data for normalized Ca^2+^ transient amplitude. (d) Pooled data for Ca^2+^ transient time to 50% decay (T50%). (Black, control group; white, MS group) ^∗^*p* < 0.05 versus control; ^#^*p* < 0.05 versus ISO. Basal conditions: *n* = 14 cells/3 animals; 100 nM ISO: *n* = 12 cells/3 animals.

**Figure 6:**
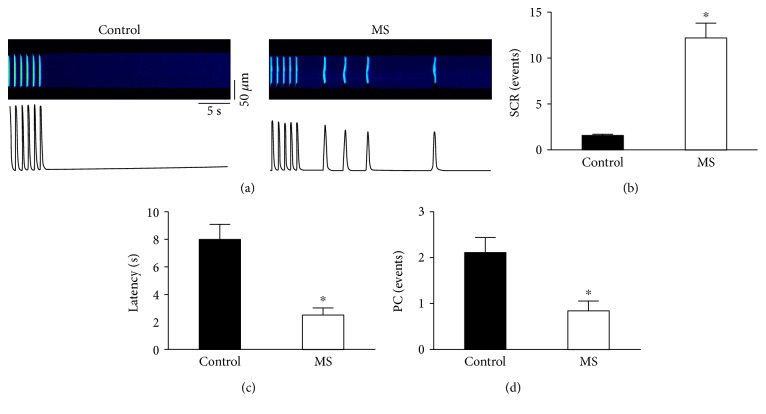
Cardiomyocytes from healthy rats show arrhythmic events after exposure to MS rat serum. (a) Representative recording of spontaneous calcium release (SCR) events for cells from healthy rats after 24 hrs exposure to control rat serum or MS rat serum under *β*-adrenergic stimulation (100 nM ISO). (b, c, d) Pooled data for SCR events and (c) partial contraction events. (Black, control group; white, MS group) ^∗^*p* < 0.05 versus control. Control: *n* = 13 cells/3 animals; MS: *n* = 5 cells/3 animals.

Similar to other groups, we determined that isolated cardiomyocytes from MS animals show decreased contractility and a slower cytosolic calcium reuptake, reflected as prolonged calcium transients [27]. Furthermore, under ISO treatment, we observed an increase in SCR and nonsynchronized Ca^2+^ release in MS cells when compared to controls (Supplemental Figure 2) [21]. However, in this work, we hypothesize that proinflammatory mediators in serum can modulate Ca^2+^ handling in ventricular cardiomyocytes acting as a trigger of arrhythmias in MS. In this regard, intracellular Ca^2+^ handling was studied in normal rat heart ventricular myocytes exposed to 20% MS rat serum for 24 h. When under 100 nM ISO stimulation compared to controls, MS serum-treated cells showed a reduced response on cell shortening, a 20% reduction in Ca^2+^ transient amplitude and a 48% increase in time to 50% decay (*T*_50%_) (Figure 5). Since more time was needed to reach resting conditions in MS serum-treated cells, the mechanisms related to calcium-induced arrhythmic events were studied. The SCR in MS serum-treated cells were five times higher (Figure 6). Hence, MS proinflammatory serum is sufficient to promote altered intracellular Ca^2+^ handling and SCR that might result in arrhythmias.

## 4. Discussion

In this study, we show that sugar diet feeding in rats promotes alterations resembling MS. MS group animals developed significant weight gain, visceral fat accumulation, elevation of mean arterial pressure, higher plasma triglycerides, and higher proinflammatory cytokine levels, similarly to results in previous studies [21, 24, 32]. Furthermore, MS group developed cardiac dysfunction associated with ventricular fibrillation and susceptibility to deadly arrhythmias during sustained adrenergic stimulation. Lastly, we identified that serum mediators from MS rats reproduced the primary Ca^2+^ handling alterations reported previously [21]. Several works have shown that intracellular Ca^2+^ mishandling is a preceding event to ventricular arrhythmias [21]. Impaired Ca^2+^ transport creates a predisposition to spontaneous, nonsynchronized Ca^2+^ release from sarcoplasmic reticulum, which in turn activates a transient inward current that is largely carried by the Na^+^/Ca^2+^ exchanger (NCX) and is the dominant inward current, triggering delayed afterdepolarizations [33]. Moreover, CaMKII-dependent phosphorylation of RyR increases sarcoplasmic reticulum Ca^2+^ leak and the susceptibility to cardiac arrhythmias [34]. In this regard, in a murine model of MS, it was observed that CaMKII is activated under prooxidant conditions and that these animals were prone to ventricular arrhythmias [21].

Despite increased intra-abdominal fat, MS rats presented no changes in heart weight. Of interest, muscle mass reduction was observed, similarly to the pathological state known as sarcopenic obesity in some metabolic dysfunction states associated with MS and atherosclerosis [35, 36]. Work by Gonçalves et al. described cardiac alterations in a similar MS rat model when feeding them with a high caloric diet [32]. At six weeks, their model showed cardiac hypertrophy and fibrosis associated with diastolic dysfunction and increased serum inflammatory cytokines [32]. The increased content of adipose tissue during sugar diet feeding might be linked to the transcriptional activation of lipid biosynthetic enzymes [37, 38], disturbing cellular function, as it happens in many organs including the heart [16, 39]. Conversely, reduction in fatty acid accumulation by nutritional, physical, or surgical interventions improves cardiac metabolism and prevents heart failure [40–42]. The clinical impact of such changes revealed the fact that, for each one-unit increment in body mass index (BMI), the risk of heart failure might increase by 5% and 7% in men and women, respectively [43]. In fact, increase in BMI correlates with a 30–100% increase in risk of heart failure [44, 45]. Regarding the association of arrhythmic effects and obesity in humans, adults with obesity were 33% more likely to have ectopic ventricular arrhythmias; for each 1 kg/m^2^ increase in BMI, there was a significant 4% increased adjusted risk for exercise-induced ventricular arrhythmias [46]. In addition, obese subjects had an increased frequency of PVC compared to healthy controls, unrelated to ventricular hypertrophy [47]. Frequent ventricular ectopy during exercise predicted a 1.8 increased risk of death and a 2.4 risk of death during the recovery phase [48]. Together, sucrose diet-induced promotion of adiposity might be a potential mediator of altered cardiac physiology. Adiposity during early stages of obesity induces chronic low-grade systemic inflammation, promoting ventricular dysfunction [15]. Inflammation is associated with macrophage infiltration and cytokine production in different organs, including adipose tissue [10]. Our results support these findings by showing that adipose tissue accumulation in MS rats leads to higher levels of TNF-*α*, IL-1*β*, and IL-6 in plasma, associated with upregulation in IL-1*β* and IL-6 gene expression in heart and visceral fat, respectively. While we have not analyzed the molecular mechanism leading to heart inflammation during MS, experimental data has shown that Toll-like receptor 4 (TLR4) stimulation by fatty acids promotes TNF-*α*, IL-6, and IL-1*β* gene expression [49, 50]. Of interest, IL-1*β* signaling has also been reported to be upregulated in hypertrophied hearts [51, 52]. In fact, IL-6 and IL-1*β* are positive modulators of insulin resistance in adipose tissue and the heart during obesity [10, 14], which might be of medical interest. Chronic inflammation has been demonstrated to play an important role in the development of insulin resistance in humans, which triggers the associated comorbidities of MS, such as dyslipidemia, hypertension, and a prothrombotic state [53]. Together, these results show a correlation of fatty acid accumulation and proinflammatory cytokine production in obesity induced by sugar diet feeding.

High levels of proinflammatory cytokines, including TNF-*α*, IL-6, IL-1*β*, and IL-2, have a profound effect on pathogenesis and prognosis in heart failure [15–18]. Our data adds a new piece of evidence by showing that high IL-1*β*, TNF-*α*, and IL-6 levels in MS rats correlate with disruption under high workloads (as during ISO treatment), maximal MVO2, and increased susceptibility to developing deadly arrhythmias. This is consistent with hearts from MS rats that show impaired electrical and mechanical activity, mainly when subject to high workloads, and that develop a more drastic reperfusion injury [24, 54]. Experimental evidence shows that IL-1*β* induces cardiac fibrosis and hypertrophy [55] and depresses cardiac function [56, 57]. The mechanism by which decreased contraction velocity is developed in MS rats has been described to be nitric oxide-dependent [58], and this might indirectly impact mitochondrial respiration performance and peak Ca^2+^ homeostasis. Additionally, cardiac modulation by cytokines is known to increase oxygen consumption with lower performance, depress Ca^2+^ transient, and impair *β*-adrenergic response [58]. A recent work has shown that TNF-*α* and IL-1*β* directly modulate Ca^2+^ handling [20]. Indeed, incubation of cardiac myocytes with TNF-*α* and IL-1*β* increased Ca^2+^ leak from sarcoplasmic reticulum and led to proarrhythmogenic events in rat ventricular myocytes [20]. Our results also show that treatment for 24 h of normal rat ventricle myocytes with 20% MS rat serum was sufficient to promote altered intracellular Ca^2+^ handling. SCR in MS serum-treated cells were five times more frequent and appeared 75% earlier under ISO stimulation. All these findings contribute to explaining the role of inflammation in heart failure by showing an increased incidence of ventricular arrhythmias in MS hearts, which we propose to be associated with impaired Ca^2+^ handling. Lastly, other animal models of obesity show Ca^2+^ dysregulation associated with intracellular lipid accumulation [59]. Our findings might have clinical relevance since it has been demonstrated that, in humans, higher levels of IL-6 have been associated with MS and sustained ventricular tachycardia and ventricular fibrillation and have also been observed in patients with malignant ventricular arrhythmias and with a number of arrhythmic episodes [60]. Patients with MS also have a greater dispersion of ventricular repolarization time and increased frequency of PVC compared to healthy controls. Furthermore, in patients with MS, outflow tract premature ventricular contractions might be due to a sympathetic over activity state, which may be the inciting mechanism [61].

An important limitation of this study is that abnormalities in Ca^2+^ handling in cardiomyocytes treated with MS rat serum were not neutralized with serum blockers (blocker antibodies, for instance) potentially responsible for cellular impairment. However, MS serum contains a number of active proteins; the dissection of individual components for further identification of the bioactive mediators in serum is important and will be the focus of our future research.

## 5. Conclusions

In summary, these findings show that the proinflammatory state in serum, adipose tissue, and the heart in MS rats correlates with decreased contractility, *β*-adrenergic signaling, and increased ventricular arrhythmias. Since markers of systemic inflammation have been found in MS, heart failure, and even ventricular arrhythmias, cardiac events occurring in subclinical states of heart dysfunction might be partially triggered by inflammatory mechanisms. Although these results cannot be extrapolated to humans, clinical implications might be elucidated, since strong correlations have been observed between higher concentrations of proinflammatory cytokines and MS and sustained ventricular tachycardia and ventricular fibrillation, which are life-threatening arrhythmias. Thus, measurement of cytokines such IL-6 and IL-1*β* may be useful during the stratification of arrhythmic risk and clinical decision making.

## Supplementary Material

Supplementary Material.

## Figures and Tables

**Table 1:** 

	Control	MS	*p*
Body weight (g)	445 ± 11	511 ± 15	0.001
Visceral adipose tissue (g)	9.1 ± 0.8	23.9 ± 2.3	<0.001
Heart to body weight ratio (mg/g)	5.6 ± 0.30	5.1 ± 0.26	NS
Triglycerides (mg/dL)	103 ± 7	176 ± 13	<0.001
Mean arterial pressure (mmHg)	113 ± 2	125 ± 4	0.012
Heart rate (beat/min)	408 ± 16	407 ± 12	NS

Pooled data for rats from both experimental groups. Control group: *n* = 19; MS group: *n* = 23.

**Table 2:** 

Cytokine	Control (pg/mL)	MS (pg/mL)	*p*
TNF-*α*	32 ± 1	59 ± 5	<0.001
IL-1*β*	386 ± 39	2972 ± 267	<0.001
IL-6	64 ± 2	90 ± 2	<0.001
IL-10	637 ± 64	661 ± 79	NS
LEP	1865 ± 271	3764 ± 358	<0.001

MS induces higher serum levels of inflammatory cytokines. Pooled data for serum cytokines from both groups at the end of treatment. Values are means ± SEM. Control group: *n* = 8; MS group: *n* = 11.
