# Supplementary material for: Proinflammatory Cytokines Are Soluble Mediators Linked with Ventricular Arrhythmias and Contractile Dysfunction in a Rat Model of Metabolic Syndrome
Source: Oxid Med Cell Longev. 2017 Oct 19;2017:7682569. doi: 10.1155/2017/7682569 (PMC5671748; doi:10.1155/2017/7682569)
Supplement: Supplementary file 1 — Supplementary Material. [file 7682569.f1.docx]

Proinflammatory cytokines are soluble mediators linked with ventricular arrhythmias and contractile dysfunction in a rat model of metabolic syndrome. (Supplemental Material)

**Supplemental Table 1**

Probes used in qPCR experiments.

**Supplemental Figure 1**

**Supplemental Fig. 1.** Gene expression of cytokines and their receptors from adipose tissue show important increase in IL6 and leptin receptor. A, B: Pooled data for gene expression from adipose tissue for cytokines (A) and corresponding receptors (B). (Black control group; White MS group) *p < 0.05 vs control. Control group: n = 6-8; MS group: n = 6-8.

**Supplemental Table 2**

Echocardiographic evaluation of cardiac function shows slight diastolic dysfunction. Pooled data for echocardiographic parameters from both groups at end of treatment. Values are means ± SEM. Control group: n = 8; MS group: n = 11.

**Supplemental Figure 2**

**
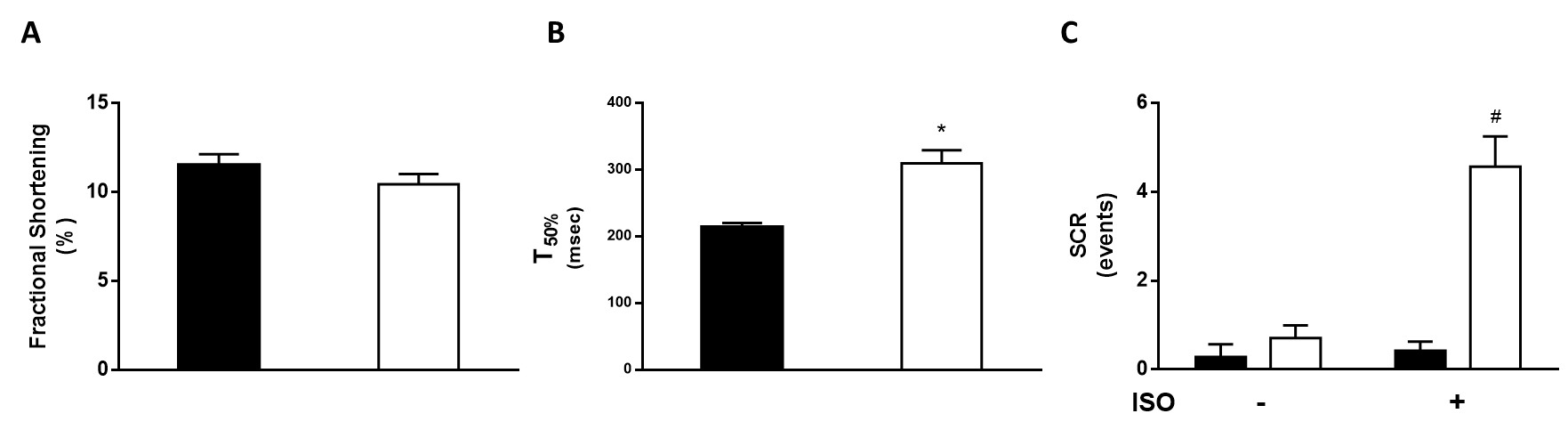
**

**Supplemental Fig 2.** Cardiomyocyte function of primary cells isolated from control and MS groups. A: pooled data for fractional shortening (%). B: pooled data for Ca^+2^ transient time to 50% decay (T50%). C: pooled data for spontaneous Ca^2+^ releases (events) under basal conditions and upon β-adrenergic stimulation (100 nM ISO). (Black control group; White MS group) *p < 0.05 vs control; # p < 0.05 vs ISO. Control group: n ≥ 6 cells/3 animals: MS group: n ≥ 8 cells/3 animals.
